# Evaluation of dose calculation accuracy of treatment planning systems at hip prosthesis interfaces

**DOI:** 10.1002/acm2.12060

**Published:** 2017-03-20

**Authors:** David Paulu, Parham Alaei

**Affiliations:** ^1^ Department of Radiation Oncology University of Minnesota Minneapolis MN 55455 USA

**Keywords:** algorithms, prosthesis, treatment planning

## Abstract

There are an increasing number of radiation therapy patients with hip prosthesis. The common method of minimizing treatment planning inaccuracies is to avoid radiation beams to transit through the prosthesis. However, the beams often exit through them, especially when the patient has a double‐prosthesis. Modern treatment planning systems employ algorithms with improved dose calculation accuracies but even these algorithms may not predict the dose accurately at high atomic number interfaces.

The current study evaluates the dose calculation accuracy of three common dose calculation algorithms employed in two commercial treatment planning systems. A hip prosthesis was molded inside a cylindrical phantom and the dose at several points within the phantom at the interface with prosthesis was measured using thermoluminescent dosimeters. The measured doses were then compared to the predicted ones by the planning systems.

The results of the study indicate all three algorithms underestimate the dose at the prosthesis interface, albeit to varying degrees, and for both low‐ and high‐energy x rays. The measured doses are higher than calculated ones by 5–22% for Pinnacle Collapsed Cone Convolution algorithm, 2–23% for Eclipse Acuros XB, and 6–25% for Eclipse Analytical Anisotropic Algorithm. There are generally better agreements for AXB algorithm and the worst results are for the AAA.

## 1 Introduction

According to Aubin^1^, there were over 500,000 hip replacement surgeries in the United States and Europe during 2005. This represents only a fraction of all prosthetic implants. Concurrently, as the mean population age continues to increase, there is a corresponding increase in the number of cancer incidences. The confluence of these two situations merits further studies to determine if proper treatment of malignant diseases in the presence of prosthetic devices is being conducted. AAPM Task Group 63^2^ points to this as a specific concern for continued treatment planning for pelvic tumor therapy when a hip prosthesis is present.

Excess dose to bone surrounding a high atomic number (z) material may have long‐term consequences including necrosis, weakening of the bone, and potential failure of the prosthetic device. Avoiding these complications may become a controlling factor in planning treatments near high‐z materials.

The effect of high‐z materials in external beam radiation therapy has been studied extensively. Das^3^ studied the backscatter dose at the interface of materials ranging from bone to lead. By comparing the dose at a reference depth in a homogeneous polystyrene block and that at the same depth with the introduction of a slab of high‐z material, backscatter dose factors (BSDFs) were calculated. The BSDF, as determined by Das, for common prosthetic device materials such as stainless steel, titanium, or cobalt‐chromium‐molybdenum, fall somewhere between 1.2 and 1.35. Erlanson^4^ studied the dose enhancement in the periphery of hip prosthesis and reported a dose enhancement of 25% at the vicinity of prosthesis.

Even though the common practice is to avoid the beams passing through high‐z prosthesis, this may not be possible in all situations. Overcoming the shadow effects of placing the prosthesis between the radiation source and the intended treatment volume may be problematic. Williams^5^ proposed a method to overcome the shadow effects by using an initial field and adding an additional boost field in the area where the prosthesis was located to deliver the intended dose to the treatment site. In addition, the treatment beams often exit through the prosthesis.

Metallic implants also present a challenge in imaging the treatment volume because they lead to poor quality CT scans. High‐z materials absorb and harden the beam which leads to streaking artifacts when the image is reconstructed. Poor quality CT images can lead to degraded diagnosis and identification of treatment planning regions of interest, and incorrect assignment of density for dose calculations. The reduction of treatment planning errors from reconstruction artifacts was studied by Bazalova.^6^ Aubin^1^ used megavoltage CT to complement standard CT images. Both sets of images were acquired and then co‐registered. Target volumes that were not readily visualized on the standard CT were able to be differentiated on the MV CT.

Whether the enhanced dose at and near the surface of prosthesis is accurately predicted by the modern treatment planning systems was the goal of this study. There have been previous studies on the accuracy of various treatment planning algorithms in the vicinity of prosthesis. Ding^7^ evaluated the accuracy of a correction‐based dose calculation algorithm in predicting the dose in the vicinity of hip prosthesis by comparing it with Monte Carlo (MC) and concluded that the algorithm underestimated the attenuation by the prosthesis. Roberts^8^ evaluated a pencil beam algorithm's accuracy when the beam passes through a prosthesis using measurements and pointed to an 11–15% overestimation of the dose by the planning system beyond the prosthesis. Lin^9^ evaluated the accuracy of a convolution‐based algorithm in predicting the dose through a prosthesis in a simple geometry by comparison with MC and concluded that it underestimates the dose upstream and downstream from the prosthesis. Keall^10^ compared MC, superposition, and pencil beam algorithms in the presence of hip prosthesis, and concluded that all of them fail in predicting backscattered dose from the prosthesis. More recently, Ojala^11^ evaluated the accuracy of Acuros XB algorithm in predicting the dose when the beam traverses a prosthesis using MC and measurements and showed a small underestimation of the dose downstream from the implant, but a larger one at the interface.

These studies often used highly reproducible volumes easily defined with good geometries and a single field to evaluate the effect of the high‐z material on dose calculation, often distal from the prosthesis. The current study focuses on two modern treatment planning systems (three dose calculation algorithms) and their ability to calculate accurate doses near the surface of a prosthetic device with multiple fields in a geometry representative of clinical cases. Three megavoltage beam energies were used in this study.

## 2 Methods

A Johnson and Johnson (DePuy) Ultima cobalt‐chromium‐molybdenum (Co‐Cr‐Mo) artificial hip prosthesis was used for this study. The AAPM TG 63 report and Hazuka^12^ detail the physical properties of several common hip prostheses. The density of Co‐Cr‐Mo is approximately 7.9 g/cm^3^ with an electron density of 6.79–6.9 relative to that of water and an effective atomic number of 27.6.

**Figure 1:**
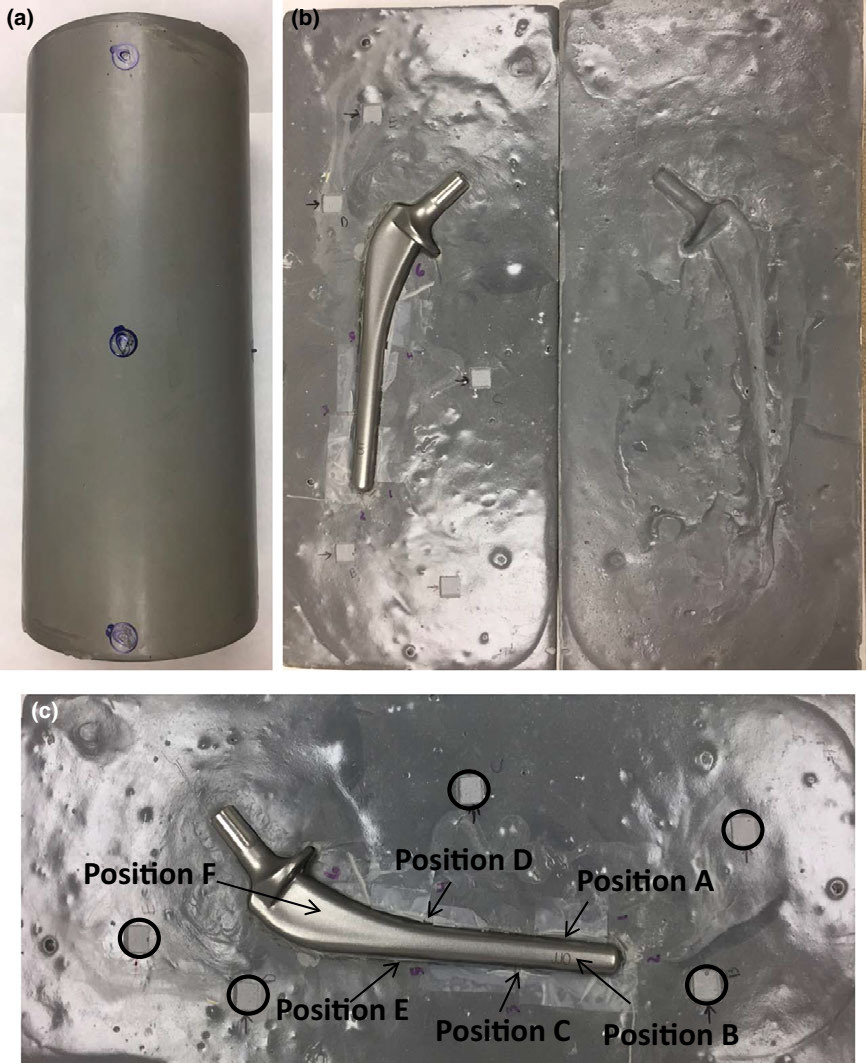
Construction of urethane phantom: (a) Phantom closed with radiopaque surface markers; (b) Phantom open with prosthesis; (c) Close‐up of TLD and OSLD locations: TLD positions are indicated with arrows, OSLD locations are indicated by circles.

A cylindrical phantom, shown in Fig. 1, was constructed of AdTech LUC4105 casting urethane. A casting urethane was chosen for its low cost and ease of molding. The initial density of the casting urethane was 1.73 g/cm^3^. The density was lowered to near that of soft tissue by the introduction of hollow lightweight glass beads. The final density of the constructed phantom was between 1.0 and 1.4 g/cm^3^.

**Figure 2:**
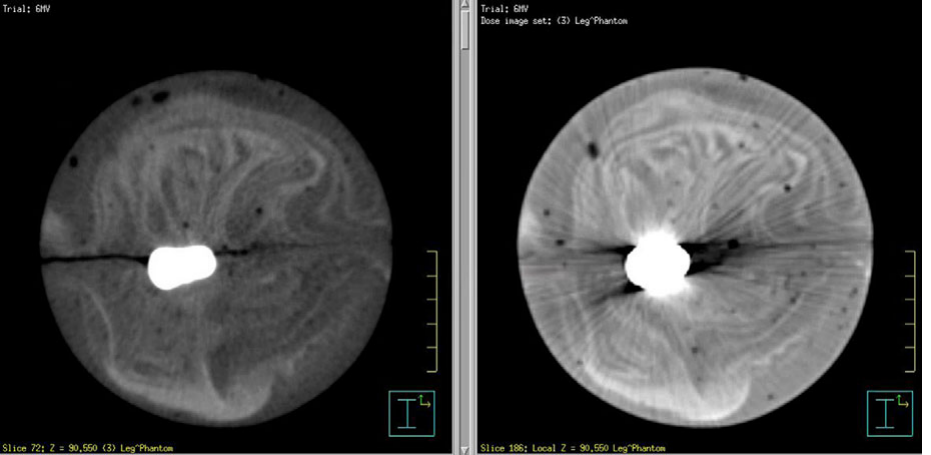
Comparison of MVCT (left) and kVCT (right) images.

The urethane was cast in hemicylindrical pieces sandwiching the hip prosthesis. This construction method allowed for easy placement and removal of the prosthesis. The final diameter of the phantom was 8.89 cm. The phantom was scanned using a kilovoltage (kV) CT scanner and the megavoltage (MV) beam of a TomoTherapy HiArt System (Accuracy, Sunnyvale, CA, USA) to eliminate streaking artifacts resulting from kV CT scan. The presence of the hip prosthesis created a significant number of streaking artifacts in the image which would have negatively impacted the ability to define the surface of the prosthesis and the TLD positions, and to calculate the dose accurately (Fig. 2). MV CT was performed using the “fine” scan setting (0.4 cm slice thickness) and radiopaque surface markers were applied to the phantom surface so the position could be reproduced on the treatment table for each run of the test.

Six thermoluminescent dosimeter (TLD) locations were selected at various points along the length of the prosthesis at the phantom interface. The TLD positions were along the length of the prosthesis but not in the same plane, so each one of them was exposed to one or more beams before they reached the prosthesis, as well as transmitted radiation from other beams. The prosthesis diameter was also different at different points: The diameter was approximately 1.4, 1.6, and 1.7 cm at points D–F, B–C, and A, respectively. Also the average depth of the TLDs relative to the beam entry points were 7.25 cm for point A, 8.25 cm for point B, 6.5 cm for point C, 5.8 cm for point D, 7.8 cm for point E, and 6.7 cm for point F (range of 4–10 cm).

Reproducible placement of the TLDs in the phantom was accomplished by carving out small volumes of the urethane and installing reference wires on two sides of the cavities to visualize the TLD locations on the CT images (Fig. 1). In order to reduce measurement inaccuracies and minimize the distance between the measurement position and the surface of the prosthesis, microcube TLDs were used (ThermoFisher Scientific, Waltham, MA, USA). Each of the LiF (TLD‐100) TLD microcubes is approximately 1 mm^3^ (1 × 1 × 1 mm). Prior to the measurements, the TLDs were annealed at 400°C for 1 h. The TLDs were then irradiated to a dose of 400 cGy between two slabs of 10 cm solid water. A Harshaw 3500 TLD reader (ThermoFisher Scientific) was used to read the TLDs. The TLDs were not annealed at a low temperature prior to analysis. Instead, the glow peaks that decay quickly were eliminated by delaying the reading by 36 h. Calibration factors were determined for individual TLDs by repeating the irradiation three times and obtaining dose/nC ratios for each TLD. Only TLDs with minimal variation between readings were used.

An additional five locations not proximal to the prosthesis were chosen for dose measurements in phantom to act as controls. The phantom was carved at these locations to insert optically simulated luminescent dosimeters (OSLD) or nanoDots (Laundauer, Glenwood, IL, USA).

The MVCT scans were exported to a Pinnacle v9.8 (Philips Medical System, Milpitas, CA, USA) and an Eclipse v11 (Varian Medical Systems, Palo Alto, CA, USA) treatment planning systems. A MVCT CT‐to‐density curve was previously created and entered into both systems. The instability of MVCT CT‐to‐density curves of TomoTherapy units has been subject of investigations but one recent publication points to its stability post‐2011.^13^ The TomoTherapy unit used for scanning the phantom is subject to the weekly calibration and monthly evaluation of the CT‐to‐density curve as recommended by Accuray and AAPM Task Group 148.^14^

Treatment plans were created in both systems delivering 400 cGy to the isocenter using 6, 10, and 18 MV photons utilizing four fields. The dose grid resolution was set to 0.1 cm^3^ in both planning systems. As the CT number of the prosthesis is outside the standard Hounsfield unit range on CT scans, the prosthesis was assigned a density of 7.9 g/cm^3^. The TLD positions were delineated in the images based on the localization wires with both points of interest (POI) and regions of interest (ROI). The volume of regions of interest was approximately 0.02 cc, which is intentionally larger than that of a TLD microcube in order to account for the dose gradient in the volume encompassing the dosimeter. The mean and standard deviation of ROI doses were obtained and compared to POI doses to detect any excess dose gradient.

**Figure 3:**
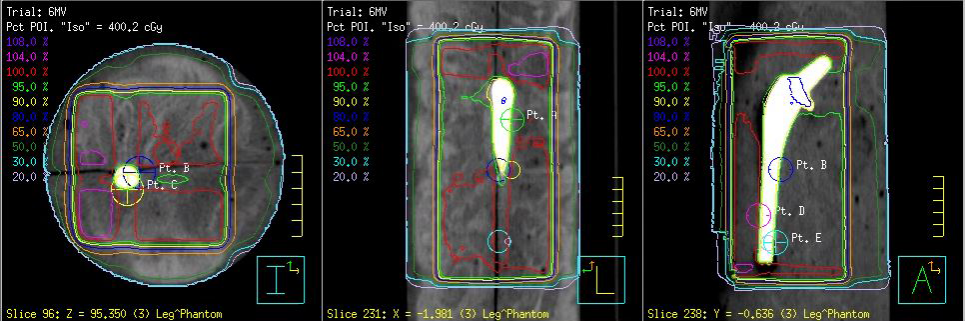
Axial (left), sagittal (center) and coronal (right) views of the four field 6‐MV treatment plan indicating TLD locations in Pinnacle.

Dose calculations were performed using collapse cone convolution (CCC) algorithm in Pinnacle and both analytical anisotropic algorithm (AAA) and Acuros XB (AXB) algorithm in eclipse. The dose distribution from one of the Pinnacle treatment plans is shown in Fig. 3.

The plans were delivered to the phantom using an Elekta Synergy linear accelerator (Elekta). The TLDs were positioned prior to delivery in the phantom and promptly removed following irradiation. Each plan was delivered to the phantom three times. As with the determination of calibration factors, the TLDs were read approximately 36 h post irradiation.

The plans were also delivered to phantom with the OSLDs in place to compare measured and calculated doses at points far from interface. In addition, a mixed energy (6, 10, 18 MV) AP/PA plan was also generated and delivered to the phantom with the OSLDs in place. This plan served as a control one as the beams did not traverse the prosthesis. The OSLDs were read after approximately 30 min using a MicroStar InLight reader (Landauer). Due to dose dependence of OSLDs, three sets of calibration curves, a low dose, high dose, and an ultra high one, were created to convert the emitted light to dose.

## 3 Results

**Figure 4:**
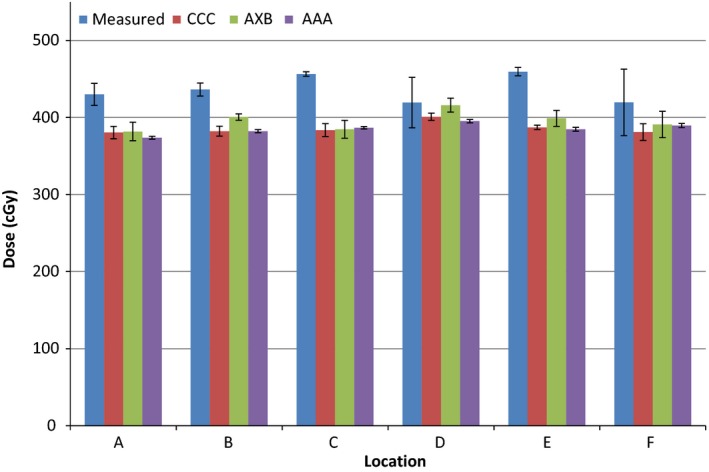
Measured and calculated doses using the three algorithms for 6 MV photons. Error bars represent standard deviations obtained from repeat measurements and regions of interest.

**Figure 5:**
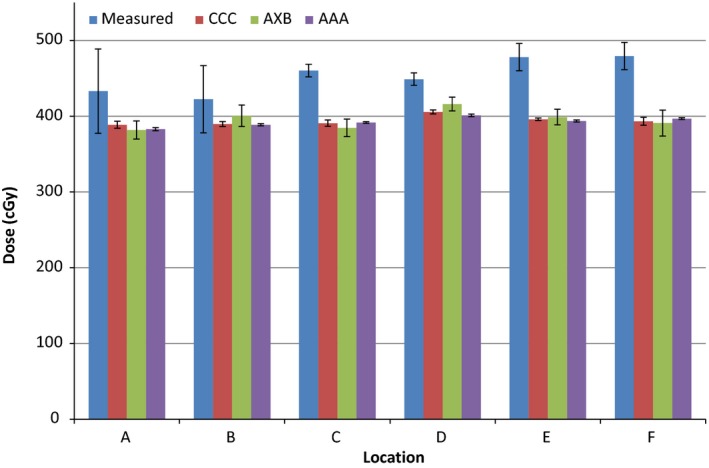
Measured and calculated doses using the three algorithms for 10 MV photons. Error bars represent standard deviations obtained from repeat measurements and regions of interest.

**Figure 6:**
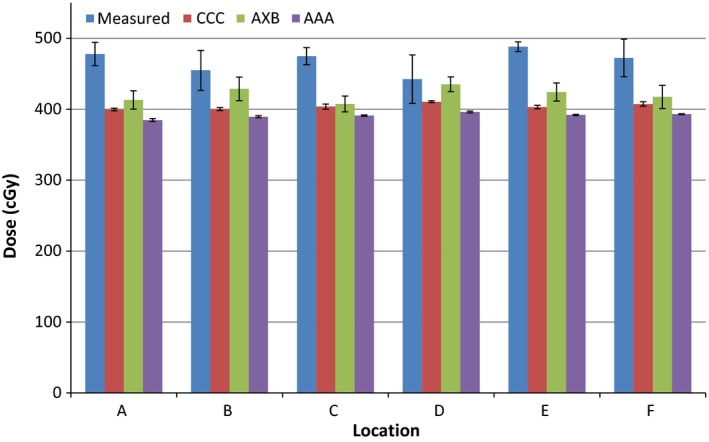
Measured and calculated doses using the three algorithms for 18 MV photons. Error bars represent standard deviations obtained from repeat measurements and regions of interest.

The calculated and measured doses and their percentage differences are shown in Tables 1, 2, 3 and Figures 4, 5, 6 for the three energies. The TPS‐calculated values are mean doses within the regions of interest. Evaluation of POI vs. mean ROI doses did not indicate any large differences or large standard deviations within each ROI. The error bars in Figures 4, 5, 6 represent standard deviations obtained from repeat measurements on the “measured” columns, and from TPS dose data within regions of interest for the other columns.

**Table 1:** Calculated and measured doses for 6 MV photons. Percentage difference: ((Measured‐Calculated)/Calculated) *100. All calculated doses are mean dose within respective ROIs

Position	Calculated dose			Measured dose	Percentage difference		
	Pinnacle CCC	Eclipse AXB	Eclipse AAA		Meas./Pinnacle	Meas./eclipse AXB	Meas./eclipse AAA
A	380.4	381.8	373.7	429.99	13.04	12.62	15.06
B	382.2	400.6	382.3	436.28	14.15	8.91	14.12
C	383.6	384.6	386.6	456.48	19.00	18.69	18.08
D	401.0	416.0	395.3	419.44	4.60	0.83	6.11
E	387.2	398.9	384.7	459.59	18.70	15.21	19.47
F	381.0	391.0	389.5	419.61	10.13	7.32	7.73

**Table 2:** Calculated and measured doses for 10 MV photons. Percentage difference: ((Measured‐Calculated)/Calculated) *100. All calculated doses are mean dose within respective ROIs

Position	Calculated dose			Measured dose	Percentage difference		
	Pinnacle CCC	Eclipse AXB	Eclipse AAA		Meas./Pinnacle	Meas./eclipse AXB	Meas./eclipse AAA
A	388.7	381.8	382.9	433.09	11.42	13.43	13.11
B	389.6	400.6	388.7	422.48	8.44	5.46	8.69
C	390.8	384.6	391.6	460.22	17.76	19.66	17.52
D	405.6	416.1	401.1	448.92	10.68	7.89	11.92
E	395.9	398.9	393.6	478.01	20.74	19.83	21.45
F	393.3	391.0	396.7	479.36	21.88	22.60	20.84

**Table 3:** Calculated and measured doses for 18 MV photons. Percentage difference: ((Measured‐Calculated)/Calculated) *100. All calculated doses are mean dose within respective ROIs

Position	Calculated dose			Measured dose	Percentage difference		
	Pinnacle CCC	Eclipse AXB	Eclipse AAA		Meas./pinnacle	Meas./eclipse AXB	Meas./eclipse AAA
A	399.6	413.1	384.7	477.85	19.58	15.67	24.21
B	400.3	428.7	389.5	454.85	13.63	6.10	16.78
C	403.7	407.5	391.1	474.83	17.62	16.52	21.41
D	410.5	435.1	396.1	442.30	7.75	1.65	11.66
E	403.2	424.3	392.0	488.14	21.07	15.05	24.53
F	407.4	417.4	393.1	472.29	15.93	13.15	20.15

The results indicate a 5–22% higher measured than calculated dose near the surface of the prosthesis for all energies for Pinnacle CCC (mean: 14.8, standard deviation: 5.1); 2–23% for Eclipse AXB (mean: 12.3, standard deviation: 6.4); and 6–25% for Eclipse AAA (mean: 16.3, standard deviation: 5.6) algorithms. Comparing measured values to TPS‐calculated ones per energy, there is a slight increase in percentage difference (averaged over all algorithms) as the energy is increased: 12.4% for 6 MV, 15.2% for 10 MV, and 15.7% for 18 MV. However, this does not mean there is a strong correlation with energy for any of the algorithms and all of them underestimate the dose at the interface irrespective of energy. As far as this study is concerned, there are generally better agreements for AXB algorithm and the worst results are for the AAA algorithm. There are obvious variations in the measured vs. calculated doses for different points, with point D having a generally better agreement than others, and point E having the worst agreement. There is no correlation between these results and the dose gradient as the standard deviations within all ROIs are very similar. There is also no correlation with phantom geometry or beam orientation.

The OSLD measurements for the four field plans indicate agreement between measured and calculated doses generally within 10% for the four field plans and 5% for the AP/PA one.

## 4 Discussion

Previous work in this area points to underestimation of the dose in the vicinity of the prosthesis by the treatment planning algorithms, which is the same as our observation. Most of the publications in this area involved MC simulations of the dose at the interface and comparison to TPS calculations, primarily limited to one energy and one TPS algorithm. This work presents measurements in a phantom mimicking a leg and evaluates three beam energies and three common commercial dose calculation algorithms.

There are several publications evaluating at the interface dose at the prosthesis. For example, Ding^7^ employed MC to evaluate the dose at the interface of stainless steel rods (density: 7.9 g/cc) for 18 MV x rays, pointing to a 15% dose increase in tissue at the metal interface, and its underestimation by correction‐based algorithm employed by CADPLAN TPS (Varian). Lin^9^ compared MC to Pinnacle's convolution‐superposition algorithm and found a 25% underprediction of dose at the surface by the algorithm. This study was limited to 6 MV beam but included two types of prosthesis: Co‐Cr‐Mo and titanium (density: 4.34 g/cc).

Keall 10 compared MC to convolution and pencil beam algorithms in calculating the dose in a phantom with iron (density: 8.0 g/cc) or titanium (density: 4.5 g/cc) blocks using a 6 MV beam. It concluded that neither algorithm predicts the increase in backscattering. Finally, Ojala^11^ compared MC with AXB and AAA algorithms for a 6 MV beam and titanium implant and showed an 8–10% underestimation of the dose by the AXB algorithm as compared to MC at the interface.

The current study shows a consistent underestimation of the dose by all three algorithms, with AXB having the best agreement, which could correlate to the inability of the algorithms to predict the backscattered dose accurately. This observation agrees with the conclusions of above references that the dose enhancement at the interface is not predicted accurately by non‐Monte Carlo‐based treatment planning systems. The prosthesis used here is similar to the ones used by three of the above references. Two of these references also studied titanium prosthesis and an additional reference used titanium only and all of them point to underprediction of the dose at the interface by the TPS algorithms, so it is fair to say that this effect is not highly dependent on the density of the prosthesis and the conclusions here can be extended to titanium implants, which have lower density.

The size of the implant should not have any effect on backscattered radiation but will, of course, affect the transmission. So the size may affect the magnitude of the dose at points downstream from the prosthesis but does not affect the underestimation of the dose due to backscatter. Should the implant be at shallow depths, which is unlikely for hip implants, the results may vary because of the uncertainty of dose calculations in the buildup region.

## 5 Conclusions

The Pinnacle^3^ treatment planning system and its CCC dose calculation algorithm, as well as Eclipse treatment planning system and its AAA and AXB algorithms were evaluated for their ability to predict the dose at the surface of a hip prosthesis within the treatment field. The results indicate that there is a consistent underprediction of the dose at the interface, irrespective of the energy and algorithm used.

The dose enhancement at the interface could lead to additional complications for patients with high‐z hip implants including premature failure of the prosthesis from bone necrosis or demineralization. With greater understanding of the dose distribution, treatment planners can make more informed decisions regarding high‐density prosthetic materials in the treatment field and potentially improve long‐term patient outcomes.

## 6 Conflict of Interest

The authors have no conflict of interest to report.
